# Prevalence of precancerous cervical lesions in women attending Mezam Polyclinic Bamenda, Cameroon

**DOI:** 10.11604/pamj.2019.32.174.16895

**Published:** 2019-04-10

**Authors:** Ngwayu Claude Nkfusai, Tchakounte Minette Mubah, Brenda Mbouamba Yankam, Tabe Armstrong Tambe, Samuel Nambile Cumber

**Affiliations:** 1 Department of Microbiology and Parasitology, Faculty of Science, University of Buea, Buea, Cameroon; 2 Department of Medical Laboratory Science, Faculty of Health Sciences, University of Bamenda, Bamenda, Cameroon; 3 Department of Nursing, Institute of Health and Biomedical Science, Cameroon Christian University Bali, Bali, Cameroon; 4 Section for Epidemiology and Social Medicine, Department of Public Health, Institute of Medicine (EPSO), The Sahlgrenska Academy at University of Gothenburg, Box 414, SE – 405 Gothenburg, Sweden; 5 School of Health Systems and Public Health Faculty of Health Sciences, University of Pretoria Private Bag X323, Gezina, Pretoria, 0001, Pretoria, South Africa; 6 Faculty of Health Sciences, University of the Free State, Bloemfontein, South Africa

**Keywords:** Precancerous lesion, cervix, age, screening, Mezam polyclinic

## Abstract

**Introduction:**

Precancerous cervical lesion is significantly a health problem globally. Thus, screening targeting women between the ages of 17-60 is being undertaken in developing countries, including Cameroon. Over 50% (7.8 per 100,000) women die of cervical cancer every year. This study was to determine the prevalence of precancerous cervical lesion, the age demography and access the risk factor.

**Methods:**

A hospital-based cross-sectional study was conducted from August 09th to October 17th 2017. A total of 60 women participated, and were screened for precancerous cervical lesion. Data were collected by using a questionnaire. Visual inspection with acetic acid and visual inspection with Lugol’s iodine was applied for the screening. SPSS version 16.0 was used for data entry and analysis. Logistic regression analysis was fitted and odds ratios with 95% confidence intervals and p-values were computed to identify factors associated with precancerous cervical cancer lesion.

**Results:**

Out of 60 study participants, 2(3.33%) were found to be positive for precancerous cervical cancer lesion.

**Conclusion:**

The prevalence of precancerous cervical lesion in women that consulted at the Mezam polyclinic is high.

## Introduction

Women in Africa as well as those residing in Cameroon, suffer a disproportionate rate of cervical cancer morbidity and mortality [1]. Cervical cancer is the second leading cause of cancer-related deaths amongst Cameroonian women in West Africa. Human papillomavirus (HPV) leads to cervical cancer. Particularly, the strains HPV 16 and 18 have been especially known to be at high risk [2]. In Cameroon, cervical cancer screening services and testing for HPV are mostly available at private health clinics and some government hospitals in the urban areas. In Cameroon most cases of cervical cancer are diagnosed at later and more serious stages. Majority of the cervical cancer victims are poor rural women who are particularly unable to access screening and testing services [2, 3]. Those who screen are unable to return for follow-up or complete treatment due to: inadequate medical advice, lack of awareness of the significance of their symptoms, cultural and religious factors as well as the lack of finances to pay for the services.

The cancer of the cervix is at the origin of 3 387 new cases and of 1000 deaths per annum corresponding to an average incidence of 8 per 100 000 person-years in 2000. It is located at the 2nd rank of female cancers in term of incidence and there thus remains a priority to public health [4]. In Cameroon one estimates that approximately 1400 to 1700 new cases of cancer of the cervix per annum and approximately 700 of them die each year [5]. The cancer of the cervix is preceded during 10 to 15 years by precancerous lesions. Those are detectable by the smear and their treatment makes it possible to avoid or reduce the risk of evolution towards an invasive cancer [6]. The screening of the cancer of the cervix in Europe (France) is individual but there are recommendations for clinical practice where it is advised to carry out a smear every 3 years after 2 normal annual smears for women from 25 to 65 years [7].

A precancerous state of the cervix is characterized by changes undergone by the cells of the collar which make them more likely to evolve in a cancer. This state is not yet a cancer, but it is strongly likely to be transformed into cancer of the cervix in 10 years or more if it is not treated in time [8]. One finds a cervical dysplasia at 1 to 5% of the women in the general population. They concern primarily the young, old women from 25 to 35 years [9]. It is estimated that each year, appear approximately 69 000 new cases of dysplasia of low rank and 15 000 cases of dysplasia of high rank in Europe [10]. The papillomavirus (human papillomavirus: HPV) are known and are very many, they are responsible for a multitude of lesions found on the skin or the mucous membranes [11]. By infecting the cells of the cervix, they lead to lesions which at term can evolve to a cancer. The co-infection by viruses AIDS and HPV is at the origin of a growing number of cancers for seropositive people. A new study on nearly a half-million patients shows it with force [12].

Five main types of cancer that affect a woman's reproductive organs are known as gynecologic cancer: cervical, ovarian, uterine, vaginal, and vulvar (Centers for Disease Control and Prevention) [13]. According to [14], of the 83,745 women diagnosed with gynecologic cancer in the United States died from the disease. CDC further stated that cervical cancer used to be the leading cause of cancer death for women in the United States. However, in the past 40 years, the number of cases of cervical cancer and the number of deaths from cervical cancer have decreased significantly because of the availability of regular Pap tests, which can locate cervical precancerous lesions before they turn into cancer. Mqoqi N *et al.* and Bailie RS *et al.* [14, 15] said cervical cancer can be prevented using: the HPV vaccines, the Pap test (or Pap smear) looking for pre-cancers, and also using Visual inspection with acetic acid (VIA), Visual inspection with Lugol’s iodine (VILI), the HPV test that looks for viruses that can cause cellular changes. Regular screening beginning at the age of 17 is the most important preventive measure. Other preventive measures include: the use of condoms during sexual contacts, limiting the number of sexual partners and avoid smoking [15].

**Figure 1:**
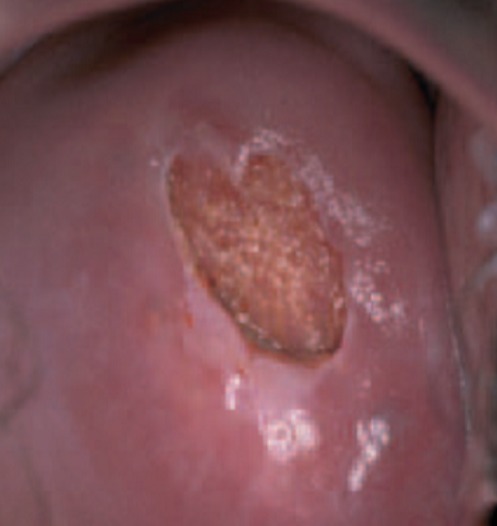
An HPV infected cervix

**Atypical squamous cells:** if the doctor tells a woman that she has atypical squamous cells, it means that abnormalities has been detected in the squamous cells of her cervix. This can indicate that she has a human papillomavirus (HPV) infection, another infection, or possibly precancerous cells of another cause. The doctor may recommend further testing to determine what the abnormalities are. In some cases, this may simply mean a repeat Pap test in a few months. A test with this kind of finding may be reported as “atypical squamous cells of uncertain significance,” abbreviated as ASCUS (Figure 1).

**Figure 2:**
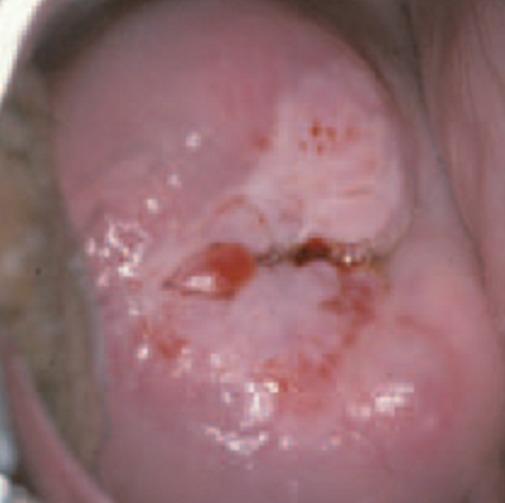
Precancerous cervical cancer infection of the cervix

**Figure 3:**
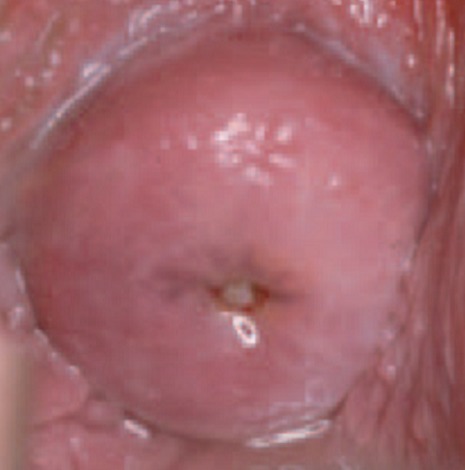
Normal cells of the cervix

**Figure 4:**
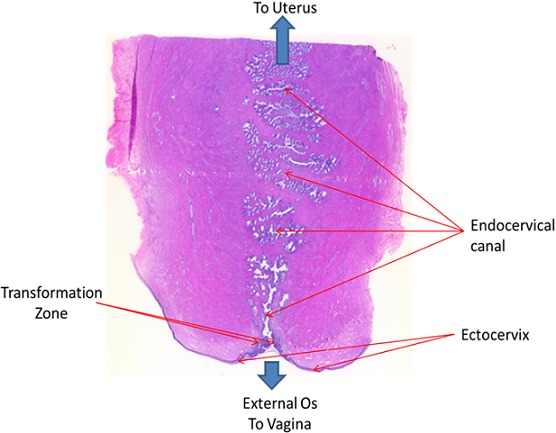
A transverse section of the cervix

**Squamous intraepithelial lesion (SIL):** this lesion means that you have changes on your cervix that may be precancerous. SIL lesions are classified as either low-grade (LSIL) or high-grade (HSIL), with high-grade lesions being more likely to progress to cervical cancer (Figure 2). **Transformation zone (TZ)** is the name given to the area of the cervix comprising epithelium that has undergone squamous metaplastic change. It is the area of squamous metaplasia between the squamocolumnar junction and the ectocervix. It is characterized by immature squamous epithelium beneath which endocervical crypts may be seen on histology. This area is where most abnormalities are thought to arise (Figure 3, Figure 4). Three vaccines Gardasil, Gardasil-9, and Cervarix have been approved by the food and drug administration (FDA) to help prevent infection with some types of HPV, including the types that cause most cases of cervical cancer. Gynecologists suggest that, boys and girls both should be vaccinated between ages 11 and 12 before they become sexually active; those between ages 13 and 26 who have not yet received the vaccine should also be vaccinated.

## Methods

### Study area, design and population

The study was carried out at Mezam polyclinic since they have the necessary facilities and the women who come are sure to be tested for and treated if need be. The study was a hospital based cross-sectional survey. The study included all women who signed the inform consent form.

### Sample size

The sample size was calculated based on the LORENTZ formula

Calculated sample size r n = t × t × p (1 - p)/ m × n

When n = required sample size; t = confidence level at 95% (standard value 1.96); m = margin of error 5% (standard value of 0.05); p = prevalence estimated of a condition under investigation in the project (3.9% of women where tested positive for precancerous cervical lesions (39).

t = 1.96, m = 0.05, p = 3.9 %( 0.1)

n = 1.96 × 1.96 × 0.039(1-0.039)/0.05×0.05

n = 3.8426 × 0.037/0.0025

n = 3.8416 × 14.8

n = 56.9.

**Sampling:** a total of 60 (due to time constrains) women were recruited for the study, using the random sampling method.

### Selection criteria

**Inclusive criteria:** only sexual active women of ages 17-60 will participate in the study. This is because this infection occurs most often in women within this age group.

**Exclusive criteria:** non-sexual active women within the said age range shall not take part in the study. This is because there is a lesser chance for non-sexual active women to come down with precancerous cervical lesions. Women who did not sign the consent form were excluded from the research work.

**Materials:** examination table, adequate light source, sterile vaginal speculum, new examination gloves, large cotton swabs, dilute (3-5%) acetic acid (vinegar) and a small bowl, lugol’s iodine solution and a small bowl, a plastic bucket with a plastic bag.

### Test procedure

### Visual inspection with 5% acetic acid (VIA)

**Figure 5:**
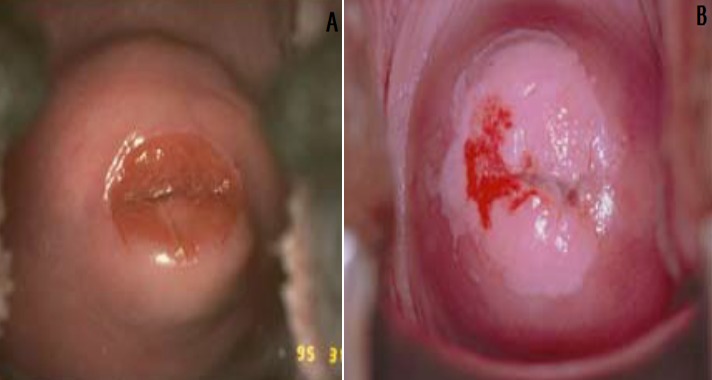
A) an acetowhite area is not significant; B) acetowhite area is significant

**Figure 6:**
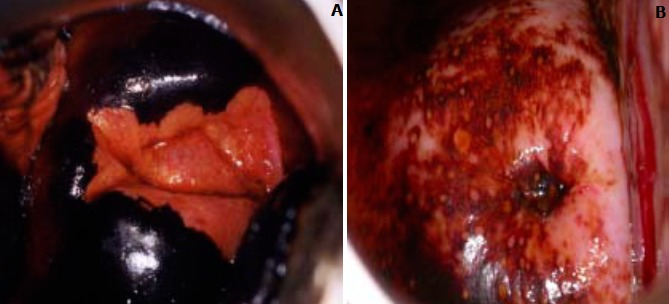
A) the squamous epithelium turns brown and columnar; B) there are scattered and uptake areas associated with immature no iodine uptake or inflammation: epithelium does not change color

**Figure 7:**
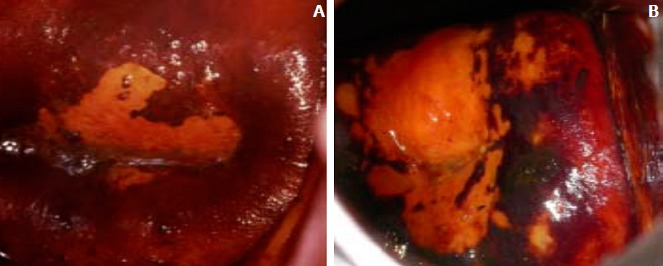
A) well-defined, bright yellow iodine non uptake areas touching the squamo-columnar junction (SCJ); B) well-defined, bright yellow iodine non uptake areas close to uptake areas touching the squamo-columnar junction (SCJ)

The woman is asked to lie in a gynecological position, and the external genitalia os examined with the help of a good light source. A speculum is used to help open the vagina and expose the external os of the cervix. A large cotton swab is deep in the bowl containing 5% acetic acid is applied to the external os. After a minute, view the cervix with the naked eyes to identify color changes on the cervix. To determine whether the test result is positive (Abnormal tissue temporarily appears white when exposed to acetic acid) or negative (no color change) for possible precancerous lesions or cancer (Figure 5). If the result is positive a confirmatory test is done using Lugol’s iodine. A large cotton swab is deep in a bowl containing Lugol’s iodine and it’s applied to the cervix. After a minute, visually observe the cervix for positive (mustard-yellow coloration) or negative (brown coloration) (Figure 6, Figure 7). Demographic and clinical data were collected using questionnaire and information from the participants were presented on tables. Data was analysed using Microsoft excel (2010) by calculating the prevalence. A record book was used to record all information about participants and later transferred and statistical calculations done.

**Ethical consideration:** an ethical approval was obtained from the institutional board of the Faculty of Health Sciences, University of Buea. An authorization was obtained from the Director of the Mezam Polyclinic and the North West Delegation of Public Health. An informed consent was signed by all participants willing to take part in the study.

## Results

Out of the 60 participants, two were screened positive for precancerous cervical lesion, and 58 were screened negative. The age range was 17-60 years with a mean age of 38.5 years.

### Demographic distribution of participants

**Marital status:** of the 60 women who participated in the study, 18 (30.00%) were singles, 29 (48.33%) were married, 5 (8.33%) were divorce and 8 (13.33%) were widows (Table 1).

**Table 1:** Distribution of participants based on marital status and age groups of participants

Marital Status		
Marital status	Number of participants	Percentage (%)
Single	18	30.00
Married	29	48.33
Divorce	5	8.33
Widows	8	13.33
Total	60	100
**Age demographic distribution based on age groups of participants**		
**Age range**	**Number of participants**	**Percentage (%)**
17-26	17	28.33
27-36	17	28.33
37-46	18	30.00
47-56	6	10.00
57-60	2	3.33
Total	60	100

**Age distribution:** out of the 60 women who enrolled, 17(28.33%) were between the age group 17-26 years, 17(28.33%) between the age groups 27-36 years, 18(30%) between 37-46 years, 6(10%) between 47-56 years, and 4 (3.33%) between 57-60 years (Table 1).

**Evaluation of risk factors:** of the 60 participants, two were screened positive within the age group of 31-45 years of age. And there were fell in the group with more than two life partners, more than 3 children, early full term pregnancy between the ages 17-20 years. One of them had a family history of cervical cancer (Table 2).

**Table 2:** Distribution of participants based on risks factors

Risk factors	Negative		Positive	
	Frequency	Percentage	Frequency	Percentage
**Age**				
17-30	24	40.00		
31-45	24	40.00	2	3.33
46>	11	18.33	0	0
**Life sexual partners**				
1	13	21.67	0	0
2>	45	75.00	2	3.33
**Parity**				
0-2	28	46.67	0	0
3>	30	50.00	2	3.33
**Full term pregnancy**				
17-20	28	46.67	2	3.33
21>	30	50.00	0	0
**Family history**				
Yes	2	3.33	1	1.67
No	57	95.00	0	0

**Prevalence of precancerous cervical lesion in women:** out of the 60 women who enrolled, 2(3.33%) where screened positive for precancerous cervical lesion and 58(96.67%) were screened negative (Table 3).

**Table 3:** Prevalence based on precancerous cervical lesion

Number of participants	Positive	Percentage (%)	Negative	Percentage (%)
60	2	**3.33%**	58	**96.67%**

## Discussion

The prevalence of precancerous lesion of the uterine cervix in Cameroon is similar to that reported in 1992 (4.2%) [16]. However, data from other countries reported a higher prevalence rate such as 16.4% women in Central African Republic Africa. In the present study, patients were not tested for HIV. In developed countries as France, the prevalence of precancerous cervical lesion rate is much lower at around of 0.5%. The difference of prevalence of the precancerous lesions of the uterine cervix from one country to another is mainly due to the existence and consistency of screening programs and management options implemented in these countries. Higher prevalence of precancerous cervical lesions in women shown by this study has been reported in South Africa (66.3%), Uganda (73%), and Zambia (76%) [17]. Studies done in Kenya and Rwanda also found the prevalence of precancerous cervical cancer to be 26.7% and 24.3% respectively, which are very high as comparable to the result of the current study. A lower prevalence of precancerous cervical cancer than the current study has also been reported from studies done in Botswana, Côte d'Ivoire, and Nigeria. In Botswana, a cervical cancer screening program of 2,175 women based on VIA found that the proportion of precancerous cervical cancer lesion confirmed by histology was 15.2% [18]. Studies recently conducted in Côte d'Ivoire and Nigeria found the prevalence of precancerous cervical cancer lesion to be 11% and 6% respectively [19]. Cervical cancer is the most common cancer in women in Africa and second to breast cancer. In Africa, it accounts for 22.2% of all cancers in women and it is also the most common cause of cancer-related death among women [20]. Knowing the prevalence and associated factors of precancerous cervical cancer lesion in women helps to take preventive measures and to know the screening requirements. In this study the prevalence of precancerous cervical cancer lesion in women was found to be 3.33% which is comparable with a previous report of Cameroon women resident in Maryland (3.9%) [21]. The high prevalence of precancerous cervical cancer lesion reveals that cervical cancer is a significant public health problem in women. Despite the high prevalence of the lesion, only four hospitals in the northwest of Cameroon were providing screening and treatment service, which significantly hampered the service accessibility to all women in the region. This could be because of the limited resources available for treatment of positive precancerous cervical cancer lesion after screening with VIA and VILI as the current limited available services are donor dependent [22].

The differences among findings of prevalence of precancerous cervical lesion in different regions of Africa could be partly due to differences in the sexual practices of the women studied [23]. Having multiple sexual partners because of cultural differences increases the risk of acquiring HPV, and in turn, the development of cervical pre-cancer and cancer. In Nigeria, with a low prevalence was reported, 96% of the study participants had two or less lifetime sexual partners. In South Africa, one of the highest reported, the median number of sexual partners was four [24]. In the present study the mean number of lifetime sexual partner is 3 and might be one of the contributing factors for the high prevalence. Unlike the findings from South Africa and Nigeria [25], though 56% of women had an average of five lifetime sexual partners, prevalence was lower than the finding in the present study, making the association unlikely. It could also be a result of the limitation of self-reported sexual practices. The other possible explanation for the different prevalences could be the differences in the study populations. The age ranged from 17 to 60 years with a mean age of 38.50 years and 85% of women were between the ages of 17-46 years. The mean number of deliveries for women with precancerous lesions of the uterine cervix was three childbirths and those with more than 3 deliveries represented 50%. Multiparity in women with precancerous cervical lesion was previously seen in Burkina Faso, West Africa [26]. These data lead to suggest the possibility of an association between multiple childbirths and cervical premalignant lesions in Cameroon.

## Conclusion

Much of the precancerous cervical lesion problem can be solved with existing or soon-to-be available technology, sufficient will, and modest resources. Early detection, screening, and treatment of precancerous cervical lesion as well as HPV will reduce the rate of cancer. Implementation of cervical cancer screening in all eligible Cameroon women should be a national priority. The prevalence of cervical lesion infection (3.33%) is high at the Mezam polyclinic Bamenda. The prevalence of women who are at risk of precancerous cervical lesion is within the age group of 31-45 years. The risk factors that play a role at mediating the disease are: age, more than one lifetime sexual partner, having more than three children as well as early full term pregnancy between the age of 17-20.

### What is known about this topic

- Prevalence of precancerous lesions of the uterine cervix in Cameroon;
- Interactive model of client health behavior and cervical cancer screening of African-American women;
- Knowledge and awareness about cervical cancer, and its prevention among interns and nursing staff in tertiary care hospitals.

### What this study adds

- The need to create awareness on the disease by sensitizing the population and screening for the disease and screening for the disease should be added as one of the screening test done during booking at antenatal;
- More health personnel’s should be trained so that they can make available the use of VIA and VILI test for screening in the district hospitals;
- The prevalence of precancerous lesions in Mezam Polyclinic, Cameroon.

## Competing interests

The authors declare no competing interests.
